# Mucosal microbiome associates with progression to gastric cancer

**DOI:** 10.7150/thno.65302

**Published:** 2022-01-01

**Authors:** Chin Wen Png, Wei Jie Jonathan Lee, Shijia Joy Chua, Feng Zhu, Gastric Consortium5, Khay Guan Yeoh, Yongliang Zhang

**Affiliations:** 1 Department of Microbiology & Immunology, and NUSMED Immunology Translational Research Programme, Yong Loo Lin School of Medicine, National University of Singapore, Singapore 117456, Singapore.; 2 Immunology Programme, Life Science Institute, National University of Singapore, Singapore 117456, Singapore.; 3 Department of Medicine, Yong Loo Lin School of Medicine, National University of Singapore, Singapore 119228, Singapore.; 4 Division of Gastroenterology and Hepatology, National University Health System, Singapore 119074, Singapore.; 5 Singapore Gastric Cancer Consortium, Singapore 119074, Singapore.

**Keywords:** gastric cancer, microbiome, intestinal metaplasia, early gastric neoplasia, Helicobacter pylori

## Abstract

**Background & Aims:** Dysbiosis is associated with gastric cancer (GC) development. However, no longitudinal study was carried out to identify key bacteria that could predict for GC progression. Here, we aimed to investigate changes in bacterial metagenome prior to GC and develop a microbiome-based predictive model to accurately classify patients at risk of GC.

**Methods:** Bacterial 16S rDNA was sequenced from 89 gastric antral biopsies obtained from 43 participants. This study was nested in a prospective, longitudinal study, whereby study participants underwent screening gastroscopy, with further 1-2 yearly surveillance gastroscopies for at least 5 years. Putative bacterial taxonomic and functional features associated with GC carcinogenesis were identified by comparing between controls, patients with gastric intestinal metaplasia (IM) and patients with early gastric neoplasia (EGN).

**Results:** Patients with EGN had enrichment of *Proteobacteria* (in particular *Proteus* genus) and depletion of *Bacteroidetes* (in particular *S24-7* family) in their gastric mucosa. Sequencing identified more patients with *Helicobacter pylori* compared to histopathological assessment, while *H. pylori* was also significantly enriched in EGN*.* Furthermore, a total of 261 functional features, attributing to 97 KEGG pathways were differentially abundant at baseline between patients who subsequent developed EGN (n = 13/39) and those who did not. At the same time, a constellation of six microbial taxonomic features present at baseline, provided the highest classifying power for subsequent EGN (AUC = 0.82).

**Conclusion:** Our study highlights early microbial changes associated with GC carcinogenesis, suggesting a potential role for prospective microbiome surveillance for GC.

## Introduction

Gastric cancer (GC) is one of the most prevalent malignant cancer worldwide and is amongst the top three leading cause of cancer-associated death globally 1. Due to the late presentation of this disease, patients with early stages of GC are often asymptomatic 2. Development of the most prevalent form of GC (i.e. intestinal subtype) follows a multistep process known as the Correa's cascade, progressing from precancerous gastritis, intestinal metaplasia (IM) to dysplasia.

Gastric carcinogenesis is the result of complex host-microbial interactions. Of which, *Helicobacter pylori* (*H. pylori*) infection remains as a major risk factor 3-5. However, *H. pylori* infection alone does not explain all cases of GC. Only approximately < 5% of infected individuals developed GC. More importantly, eradication of *H. pylori* for GC resulted in mixed outcomes, with some studies demonstrating success in halting the progression of gastritis 6-8. However, in most clinical trials, successful *H. pylori* eradication did not lead to reversal of neoplasia, or prevention of GC progression 9, 10.

Meanwhile, non-*H. pylori* microbiota was also found to play an important contributing role for development of GC. Hypergastrinaemic insulin-gastrin (INS-GAS) transgenic mice developed more severe GC in the presence of complex gastric microbiota compared to germ-free mice infected with *H. pylori* alone 11, 12. Similarly from human microbiome studies, dysbiosis of non-*H. pylori* microbiota was associated with atrophic gastritis, IM and GC when compared to either healthy controls or patients with superficial gastritis 13-15.

However, there were no prior prospective studies, with repeated paired sampling of gastric mucosa, to investigate bacterial taxonomic and functional changes prior to gastric cancer. In this study, participants were part of a larger prospective longitudinal study 16, whereby participants underwent screening gastroscopy and subsequently 1-2 yearly surveillance gastroscopy. Gastric antral biopsies from these patients, at each visit, were profiled for the mucosal-associated bacterial composition to identify putative metagenomic taxonomic and functional changes prior to GC. Here, we report the differential gastric mucosal bacterial composition of participants across varying severity of IM, as well as of patients with early gastric neoplasia (EGN). Baseline bacterial features were also used to develop a predictive model to accurately classify patients at risk of EGN.

## Methods

### Participants and clinical data

This study was nested within the Singapore Gastric Cancer Epidemiology Program, GCEP 16, 17. GCEP is a prospective multi-center longitudinal cohort, which recruited 2,980 Chinese subjects aged 50 or above, who had previous history of *H. pylori* infection and/or known premalignant gastric lesions such as atrophic gastritis and IM. All participants underwent gastroscopy by board-certified gastroenterologist and gastrointestinal surgeons using high quality white light endoscopy platforms with rapid escalation to high resolution image-enhanced endoscopy upon detection of abnormal pathology. The examination protocol was systematic with detailed photographic documentation. Participants who had never had an endoscopy or who had an endoscopy more than 12 months before the enrolment underwent a prospective baseline endoscopy. All tissues specimens were collected with ethics approval from the Singapore National Health Group Domain Specific Review Board (DSRB number: 2000/00329).

### Prospective follow-up and definition of outcomes

After the baseline endoscopy, participants were scheduled for surveillance endoscopies 1-2 yearly. At each gastroscopy visit, 6 biopsies consisting of 2 each from the antrum, corpus, 1 from the incisura and 1 from the cardia were taken at each gastroscopy examination, and graded for chronic gastritis, gastric atrophy, intestinal metaplasia (IM), and dysplasia by two independent pathologists. Severity of gastritis and IM was scored using the updated Sydney System classification. Patients with focal, mild (< 30%) IM were classified as low-risk IM, whereas multi-focal, or moderate-severe IM (≥ 30%) were consider high-risk IM. The endpoint of GCEP was EGN, which included histological diagnosis of high-grade dysplasia, intramucosal carcinoma, and adenocarcinoma. Dysplasia was graded according to the revised Vienna classification and classification of carcinoma was done according to the WHO classification of tumours and the AJCC Cancer Staging Manual, Eight Edition 18, 19. All participants were followed up annually, for at least 5 years, either at the clinic or via telephone for symptom review in the years that they were not scheduled for endoscopy surveillance. Participants who did not complete year 5 surveillance endoscopy were matched against the National Registry of Diseases Office for missed diagnoses of gastric cancer.

### Sample collection and DNA extraction/QC

One gastric antral biopsy from each patient at each gastroscopy visit was obtained for bacterial 16S metagenomic analysis. DNA from snap-frozen mucosal biopsies were extracted with the Wizard™ Genomic DNA Purification kit (Promega, USA). Approximately 5 mg of antral gastric mucosal tissue was used. The samples were overlaid with 600 µl of Nuclei Lysis Buffer (50 mM Tris-HCl, pH 8.4, 0.5% SDS, 1 mM EDTA) and homogenized for 10 seconds. An aliquot of 3 µL of RNAase A Solution was added and incubated for 15 min at 37 °C. After which, 200 µl of Protein Precipitation Solution was added. DNA was further purified using the QIAamp DNA Mini kit (Qiagen, USA) before storage at -20 °C until required.

### Bacterial 16S rRNA gene library preparation and sequencing

Bacterial 16S rRNA gene amplicon library preparation and sequencing was carried out according to Illumina “16S Metagenomic Sequencing Library Preparation” protocol. Briefly, bacterial 16S rDNA V3-V4 region was amplified using V3-V4 specific primers 20 with adapters and paired-end indices that allowed pooling and sequencing of PCR products. Amplicons were sequenced using MiSeq reagent kit V3 on Illumina MiSeq platform (Illumina, USA) that generated 2 x 300 bps paired-end reads. Demultiplex reads in FASTQ format (excluding reads that did not pass cluster filter) were used for data analysis as described below.

### Data analysis

#### OTU construction and taxonomic assignment

OTU clustering and taxonomic profiling of 16 rRNA amplicon sequencing data was performed with the UPARSE pipeline (version 11) 21. Briefly, paired-end reads from all datasets were first merged, filtered and de-replicated. For quality-filtering the UPARSE threshold of Emax = 1 was used, at which the most probable number of base errors per read is zero for filtered reads, and a truncation quality threshold of 15. Furthermore, reads were trimmed-filtered by a minimal length of 400 bp. OTUs were then sorted by size, singletons were discarded and OTUs were clustered at 97% similarity. Subsequently, the representative sequences for each cluster were mapped against the Greengenes 16S rDNA database (version 13.5) 22 to filter chimeras and obtain taxonomic assignment. This resulted in the generation of 644 OTUs, whereby 11% of the OTUs were classified at the species level, 59% genus, 87% family and 96% class. OTUs were further required to occur in at least 5% of the sample cohort, before use in differential abundance testing.

#### Statistical association analysis

Community microbial analysis were analysed using the R packages Phyloseq and Vegan 23, 24, whereby rarefaction was implemented to the depth at 90% of the minimum sample depth (3120 reads) of the dataset for diversity measurements. For prediction of functional gene content, raw OTU abundances were first normalised by their known/predicted 16S copy number prior to predicting metagenomic functional potential using the software PICRUSt2 25, and reported as functional orthologs using the KEGG orthology database, whereby they were further agglomerated into higher-order KEGG modules and MetaCYC pathways. Gene set enrichment analysis of significant KOs (P < 0.05) were carried out using clusterProfiler R package 26. For differential abundance testing of microbial features, DESeq2 27 was used to normalised raw read counts whereby a case-control study design was adopted, with samples without IM/EGN as the control. Significant differentially abundant features were expressed as log2-fold change, and accounted for multiple testing through Benjamini-Hochberg correction. To construct a prediction model for subsequent gastric neoplasia OTUs abundances were converted to log10 relative abundances, then z-normalised, before passing through a 5-repeated 5-fold cross validation Lasso logistic regression using the SIAMCAT R package 28, and performance of the prediction model is presented as an AUROC with 95% confidence interval.

## Results

### Patient cohort and samples

**Figure 1:**
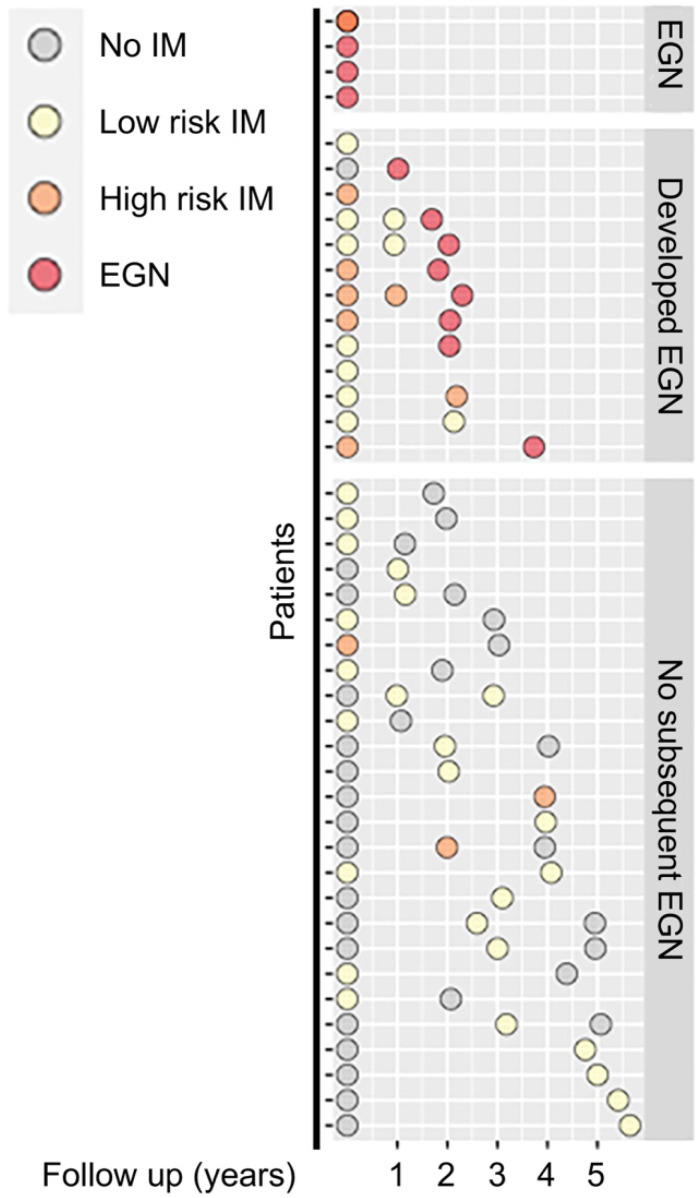
** Schematic diagram showing clinical features of patients at baseline and at subsequent follow up.** Each circle denotes a single biopsy sample from the respective patients, whereby they are coloured according to their corresponding histological diagnosis: No IM (grey), Low risk IM (yellow), High risk IM (orange) or EGN (red).

Among the 43 participants who underwent a total of 89 gastroscopies, four were found to have EGN at baseline. Participants without EGN at baseline, were then stratified by stage of the Correa's cases, (a) superficial gastritis without IM (n = 17), focal, mild IM (i.e. low risk, n = 16), or moderate-severe, multifocal IM, (high risk, n = 6). One third (n = 13/39) of the participants at baseline subsequently developed EGN (Figure 1).

The baseline characteristics of the study participants, stratified by baseline histology, are detailed in Table 1. Majority of the patients had prior exposure to *H. pylori* (n = 31/43, 72.1%). However, none of the patients had *H. pylori* present upon histopathology review. There was a greater proportion of men with IM (77.3%, n = 17/22) and the mean age amongst patients with either high-risk or low-risk IM were significantly higher than patients with no IM (p = 0.01).

### Differences in abundance of specific bacterial taxa in tissues from IM and EGN compared to no IM patients

To study the bacterial community changes associated with pre-GC stages (i.e. no IM, low risk IM, high risk IM, EGN), we assessed microbial diversity and richness of mucosal biopsy samples via the analysis of 16S rRNA gene hypervariable V3-4 regions. Overall, there were no significant differences in alpha and beta diversity across samples from different groups. Briefly, alpha diversity measurement based on various estimator including Chao1, ACE, Shannon, Simpson diversity index, did not show significant differences in species richness and phylogenetic diversity when comparing all 89 samples from across the different patient groups (Supplementary Figure 1A, 1B). Although bacterial profiles in samples from high risk IM and EGN group had larger variations compared to no IM and low risk IM (Supplementary Figure 1C), test of multivariant homogeneity of groups dispersions measured by the Bray-Curtis (*P* = 0.719) and ANOSIM based on Bray-Curtis (*P* = 0.209) did not demonstrate significant differences in beta diversity across the groups (Supplementary Figure 1D).

### Associated microbial taxonomic in GC carcinogenesis

**Figure 2:**
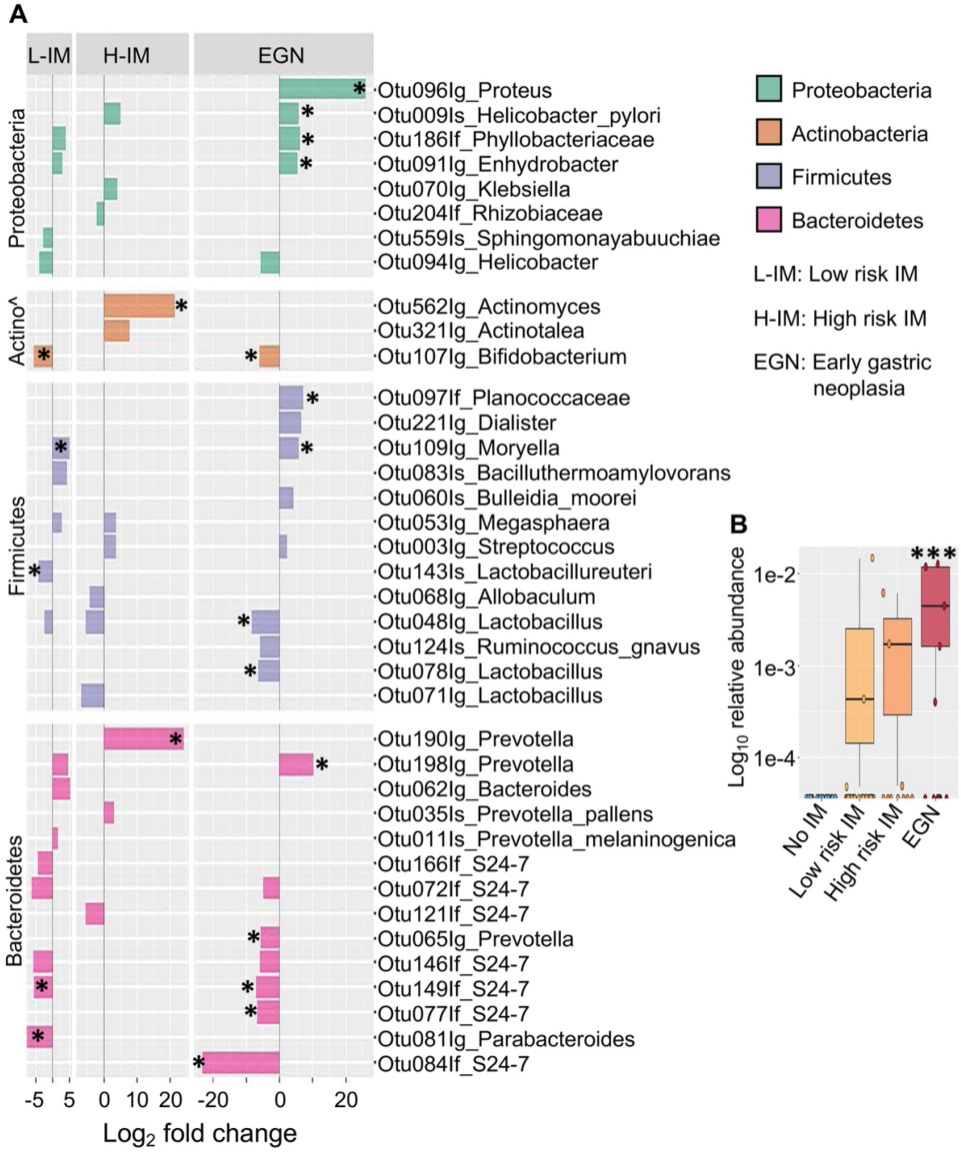
** Differentially abundant bacterial OTUs in patients with IM and EGN compared to patients with no IM.** (A) Bar chart shows the log2 fold change (X-axis) differences in abundance of top bacterial taxa in low risk IM (L-IM), high risk IM (H-IM) or patients with dysplasia (EGN) compared to No IM group based on Deseq2 univariant analysis. All comparisons *P* < 0.05, *denotes Benjamini-Hochberg adjusted P (P_adj_) < 0.1. (B) Box and whiskers plot showing increased Proteus bacteria in patients with dysplasia compared to No IM patients. ***P < 0.0007, FDR = 0.1, multivariant test (MaAsLin2).

While changes in bacterial diversity were not identified, abundances of specific bacteria taxa in patients with IM or EGN compared to those with no IM were found to be significantly different (*P* < 0.05 and Benjamini-Hochberg adjusted *P* (*P_adj_*) < 0.1). Differentially abundant OTUs across the GC carcinogenesis stages were represented by enrichment of *Proteobacteria* and depletion of *Bacteroidetes* (Figure 2A). Among the *Proteobacteria*, abundance of OTUs from the genus *Proteus* were increased by 26 (log_2_) fold (*P_adj_* < 0.0001) in samples with EGN. In addition, *H. pylori* (species) was increased in EGN and high risk IM by ~41-fold (*P_adj_* < 0.05), whereas OTUs representing *Phyllobacteriaceae* and *Enhydrobacter* were increased in both EGN and low risk IM.

In addition, OTU representing *Actinomyces* (genus) that belongs to the *Actinobacteria* phylum was increased in high risk IM (Figure 2A). In contrast, *Bifidobacteria* (genus) were reduced in both EGN and low risk IM by 62-fold (*P_adj_* < 0.1). Changes in abundance of OTUs belonging to the *Firmicutes* phylum were also detected. These included *Moryella* (genus*)*, which was increased in samples from EGN and low risk IM by ~55-fold (*P_adj_* = 0.07). *Lactobacillus* (Genus), which is a key lactic acid bacteria found in the gastric environment, was also significantly reduced by ~ 99 to 360-fold (*P_adj_* < 0.05) in EGN and low risk IM patients. Significant changes in the abundance of various OTUs belonging to the *Prevotella* genus and *S24-7* group were also detected in EGN and low risk IM group. In particular, *S24-7* group was reduced by 6.7 to 23 (log_2_) fold (*P_adj_* < 0.05) compared to no IM. Of note, *S24-7* abundance was also found to be positively associated with IM reversal and negatively associated with IM progression (Supplementary Figure 2). Upon correction for other patient significant covariates such as age, gender, *H. pylori* exposure, tissue histological characteristics and repeated measurements, abundance of *Proteus* (P = 0.0007, *P_adj_* < 0.1, FDR = 0.1) remained as the only bacterial taxonomic feature that was significantly higher in samples with EGN compared to no IM (Figure 2B).

### Specific gastric bacteria in baseline patient samples were predictive of EGN progression

**Figure 3:**
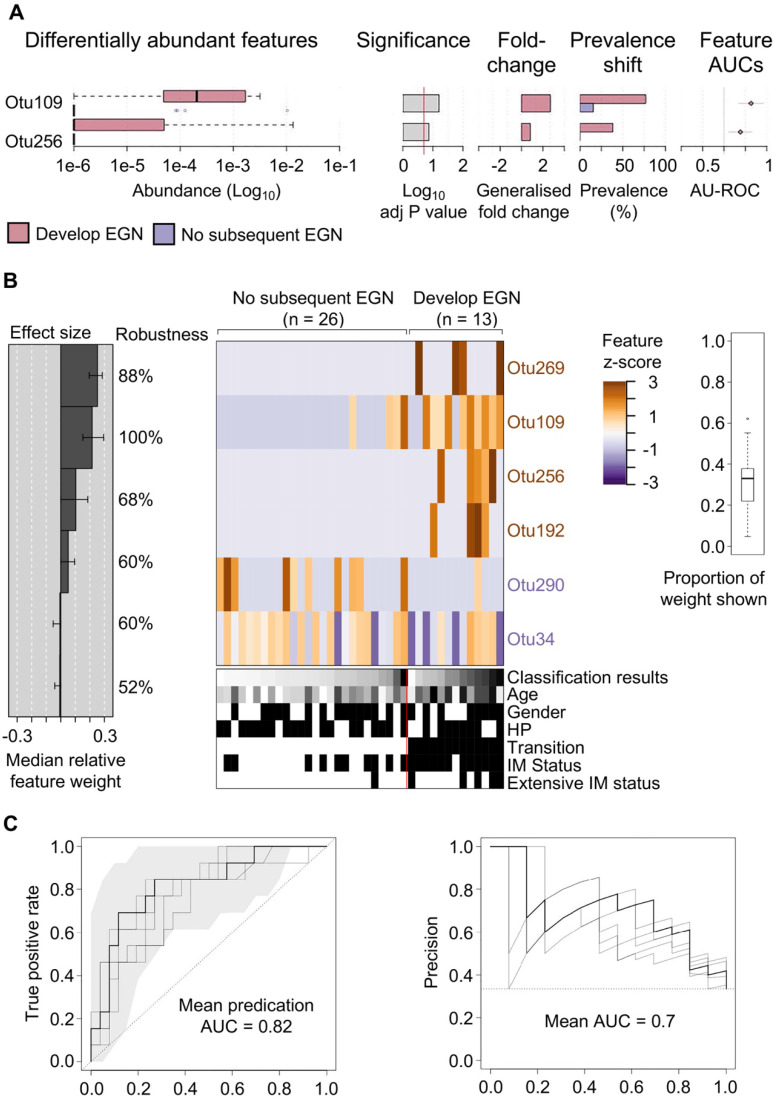
** Specific gastric mucosal bacteria are predictive of disease progression to EGN.** (A) Bar charts showing baseline OTUs (log10 relative abundance) that were significantly different between patients that progress to EGN compared to those who did not. (B) Model interpretation plots show the median relative feature weight (left barplot) of the top selected features, effect size, the robustness (percentages shown to the right of the barplot), and the feature z-scores across samples, ordered by group and classification score (right heatmap with annotations, black bar = positive status). HP denotes samples with prior clinical history of *H. pylori* infection. (C) Receiver operating characteristics (ROC) curve and precision recall curves showing the performance of the model shown in (B). SIAMCAT package was used for all analysis and calculations.

One third (n = 13/39) of study participants, without baseline EGN, were found to have EGN at the end of the 5-year study. Two microbial features demonstrated a statistically significant difference (FDR < 0.1) in the relative abundance at baseline between patients who did and did not develop EGN. Both OTU109 (*Moryella* genus) and OTU256 (*Vibro* genus) were significantly more abundant at baseline among patients who did develop EGN (Figure 3A). To evaluate if microbial taxonomic features would accurately predict GC risk, baseline microbial taxonomic features were analysed using feature-selecting machine-learning model (i.e. repeated 5-fold cross validation lasso regression). This model, which incorporated 6 taxonomic features, namely OTU109, OTU256 (described above), OTU269 (family: *Comamonadaceae*), OTU192 (genus: *Paludibacter*), OTU290 (genus: *Agrobacterium*), OTU34 (Order: *Clostridiales*) provided the highest classification power for progression to EGN, with the mean predication AUC at 0.82 for true positive and the mean AUC at 0.7 for precision recall (Figure 3B).

### Microbial functional features associated with subsequent EGN

**Figure 4:**
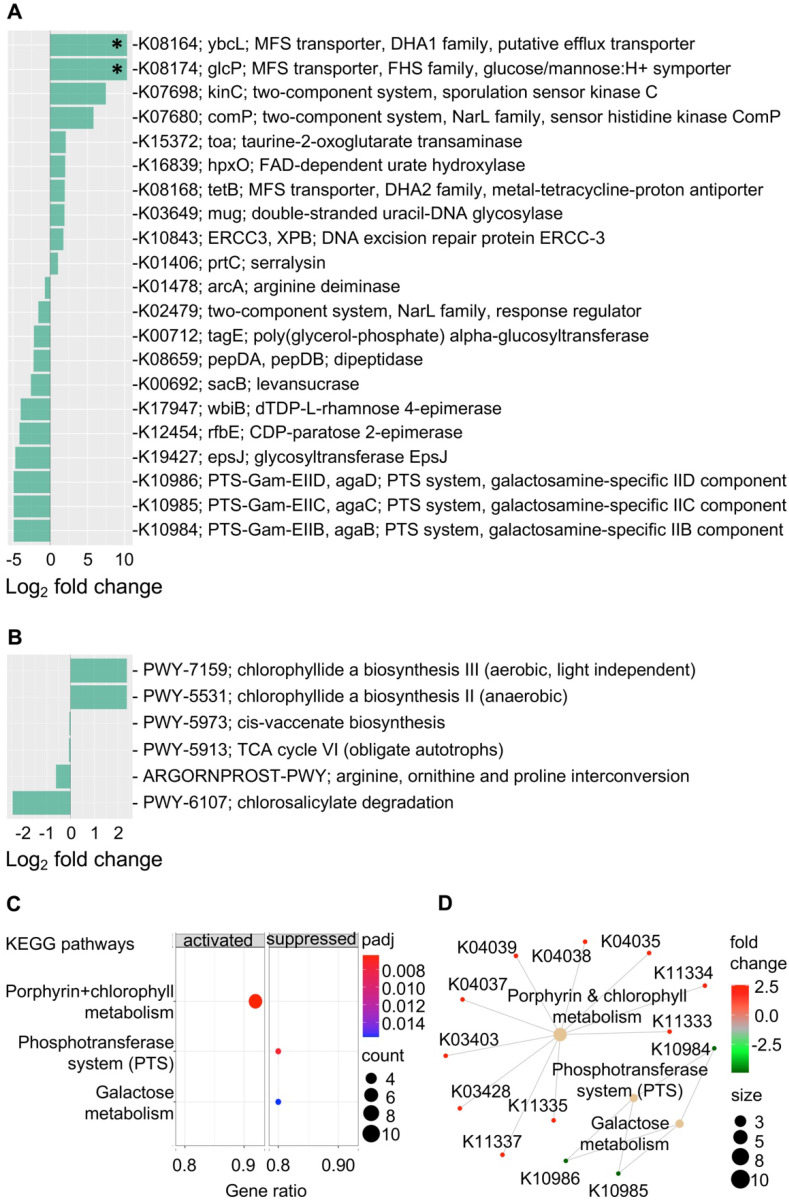
** Gastric mucosal-associated microbiome functions by PICRUSt2 based on bacterial 16S rRNA gene sequences.** Bar plots show top differentially abundant (A) KEGG orthology (KO) features (*P* < 0.005) and (D) MetaCyc pathways (*P* < 0.05) in baseline samples from patients that progress to EGN and patients that do not progress. *denote Benjamini-Hochberg *P*_adj_ < 0.01 (DESeq2). (C) Dot plot showing top enrichment KEGG pathways that were either activated or suppressed in samples from patients that progress to EGN compared to patients that do not. “padj” and “counts” denote Benjamini-Hochberg adjusted *P* values and gene counts respectively. (D) Network plot showing KOs features and linkages associated with the enriched KEGG pathways.

Gene content and predicated gene family abundance were inferred from the bacterial 16S rRNA gene sequencing data using PICRUSt2 25. In total, 261 differentially abundant KEGG orthologs (KOs) (*P* < 0.05) were identified and mapped to 97 KEGG pathways, 24 KEGG BRITE and 40 functional modules (Supplementary Data 1). Of these, the top differentially abundant KOs (*P* < 0.005) are shown in Figure 4A. These top KO entries were assigned to; (a) 19 KEGG pathways (most represented pathways included; “metabolic pathways” (38%), “galactose metabolism”, “phosphotransferase system” and “two-component system” (14% each); (b) 9 BRITE hierarchies (most represented BRITE list object included; “Enzymes” (71%), and “Transporters” (29%) and; (c) two functional modules including “M00704, tetracycline resistance” and “M00546, purine degradation” (Supplementary Data 1). Within the most represented metabolic pathways, key factors involved in galactosamine PTS system EIIB component (agaB, agaC, agaD) that are important for galactose metabolism were reduced in the progression group (Log_2_ fold change = -4.9, *P* = 0.003). Other metabolic pathway-related enzymes that were also reduced included levansucrase (sacB), arginine deiminase (arcA), CDP-paratose 2-epimerase (rfbE) and dTDP-L-rhamnose 4-epimerase (wbiB), which are involved in biosynthesis of secondary metabolites. On the other hand, taurine-2-oxoglutarate transaminase (toa) required for beta-alanine metabolism and FAD-dependent urate hydroxylase (HpxO), which catalyze the hydrolysis of uric acid for purine metabolism were increased in the progression group.

In addition, an increased abundance of KOs that is related to transcription machinery and DNA repair mechanisms were detected in the progression group. Functions represented in key KOs include the DNA excision repair protein ERCC-3, double-stranded uracil-DNA glycosylase, DNA repair protein RadD and DNA-directed RNA polymerase subunit beta-beta (Figure 4 & Supplementary Data 1). Functions related to protein kinases such as sporulation sensor kinase C and E, NarL family proteins including sensor histidine kinase ComP, nitrate/nitrite sensor histidine kinase NarQ and sensor histidine kinase NreB and sensor histidine kinase MalK from the CitB family protein were upregulated in baseline samples from patients that progressed to EGN. Of note, among the various top functional predications, increased cationic antimicrobial peptide (CAMP) resistance and vancomycin resistance were also identified in the progression group. Changes in these functions were represented by upregulation of serralysin, serralysin4-amino-4-deoxy-L-arabinose transferase (prtC), undecaprenyl phosphate-alpha-L-ara4N flippase subunit (arnF), undecaprenyl phosphate-alpha-L-ara4FN deformylase (arnD) and response regulator VanR (Figure 4 and Supplementary Data 1). At the same time, MFS transporters, namely ybcL and glcP were significantly increased (Log_2_ fold change = 10.3, *P* < 0.0001, *P_adj_* < 0.02) in the progression group.

Further KEGG pathway enrichment analysis of all 261 differentially abundant KOs showed that the interrelated phosphotransferase system (PTS) (enrichment score = -0.957, *P_adj_* = 0.006) and galactose metabolism (enrichment score = -0.904, *P_adj_* = 0.016) pathways were suppressed in the progression group, whereas porphyrin and chlorophyll metabolism were increased (enrichment score = 0.827, *P_adj_* = 0.006) (Figure 4C and 4D). In line with this, chlorophyllide, a biosynthesis pathways (PWY-7159, PWY-5531) that are important for the formation of chlorophyllide a, an intermediate for bacteriochlorophyll synthesis was overrepresented in the progression group (Figure 4B and Supplementary Data 2). Four other MetaCyc pathways that were overrepresented in the non-progression group included the (a) cis-vaccenate biosynthesis (PWY-5973) pathway required for the synthesis of the main unsaturated fatty acid cis-vaccenate; (b) TCA cycle VI (PWY-5913), a catabolic aerobic respiration reaction for the generation of energy; (c) arginine, ornithine and proline interconversion (ARGORNPROST-PWY) pathway that carries out fermentation of different forms of amino acids for growth based Stickland reactions and (d) the chlorosalicylate degradation (PWY-6107) pathway that is utilised by microbes that are able to chlorosalicylate as a carbon source for growth.

## Discussion

Components of the microbiome, in particular *H. pylori,* contribute to the initiation and propagation of GC carcinogenesis. In this study, we characterised the gastric mucosal bacterial composition through 16S rRNA amplicon sequencing, demonstrating that the gastric mucosa was predominantly composed of phyla groups *Proteobacteria* and *Firmicutes*. This finding is consistent with other reports 29, 30. In addition, *H. pylori* were also significantly enriched in patients with EGN. Although all patients included in this study were screened to be negative for *H. pylori* infection, DNA sequencing was still able to identified patients with *H. pylori*. This suggests that target sequencing to identify *H. pylori* could be applied clinically in addition to histological assessment to detect for *H. pylori* infection. Indeed, our group 17 and others have previously used PCR methods to detect *H. pylori* infection. However, most of such applications have been limited to stool analysis, with a high sensitivity of 93.8% 31. Currently we are validating a point-of-care diagnostic for *H. pylori* infection using such PCR methods, by sampling the 23S rRNA to detect point mutations for additional predictive antibiotic (i.e. clarithromycin) resistance information 32, thereby providing patients with direct-based eradication therapies.

In this study, we also illustrated important non-*H. pylori* changes across different stages in the Correa's cascade of gastric carcinogenesis. Patients further along the progression of the Correa's cascade had an enrichment of metagenomic taxonomic features belonging to the *Proteobacteria* phyla, demonstrated through feature-based regression analysis, such as *Proteus* (genus) which is a potential gastrointestinal pathobiont shown to promote intestinal inflammation 33. Other key *Proteobacteria* taxonomic features increased amongst patients with dysplastic lesions included *Phyllobacteriaceae* and *Enhydrobacter*. Changes in the abundance of these *Proteobacteria* were also previously reported to be significantly associated with GC development 14, 34.

Beneficial bacterial taxonomic features, such as lactic acid bacteria genera *Lactobacillus* and *Bifidobacteria*, were diminished in IM and EGN. *Lactobacillus* is often characterised as a transient probiotic bacteria from the oral cavity and is included in the formulation of many probiotics 35, 36. Lactic acid bacteria have also been previously associated with CRC prevention by alleviating apoptosis and antioxidant DNA damages 37. Apart from the above bacterial taxa, lower abundance of *S24-7* (or *Muribaculaceae*) of the *Bacteroidetes* phylum was also detected in IM and EGN. Recently, *S24-7* has been associated with increased ILC2 in the stomach, which in turn provides immune protection through the induction of IgA 38.

One third of patients in this study subsequently developed EGN, from which we identified a constellation of six bacterial taxonomic markers, at baseline, that accurately classify patients who would develop EGN (Figure 3). Significant predictors of EGN risk included microbial taxonomic features belonging to bacterial genera *Moryella* and *Vibrio,* which are also significantly increased at baseline amongst patients who would develop EGN. *Moryella* (genus) has been shown to exhibit specific co-occurrence with *H. pylori* in IM 15, which is consistent with our data that shows patients with either IM or EGN have both increased abundance of both *H. pylori* and *Moryella* (genus) (Figure 2). While, *Vibrio* (genus) is a known pathogen of the gastrointestinal tract 39, its association with GC pathogenesis has not been widely reported. When these two OTUs were implemented together with four other OTUs, the prediction model was able to identify patients who would progression to EGN with an accuracy of 82%. Unlike previous microbial biomarker discovery efforts for GC from cross-sectional case-control studies, we examined and compared prospectively the bacterial profiles of patients at baseline, before the development of EGN. Thus, we believe that our findings would more accurately represent putative metagenomic features associated with GC carcinogenesis. However agreements with external cohorts are required to further validate this panel of taxonomic features as a robust EGN progression biomarker.

We further investigated metagenomic functional changes associated with GC carcinogenesis. Features associated with dysregulation of nutrient metabolism were found to be significantly different between patients who developed EGN and those who did not. Patients who developed EGN had gastric microbial communities harbouring decreased abundances of agaB, agaC, agaD, sacB and rfbE, which represent reduced galactose, sucrose and starch metabolism (Figure 4A, 4C, 4D). This is in line with the observation of decreased mucin production in gastric IM 40, that could alter the nutritional content within the gastric mucosal, and in turn the composition of the gastric microbial community. In addition, patients that progressed to EGN had lower abundances of functional features that represent microbial pathways associated with arginine degradation. Arginine deprivation induced through bacterial arginine deiminase has been shown to supress growth of various tumour cell types 41-43. Hence in patients that developed EGN, a baseline reduction in microbial arginine deiminase function may suggest possible increase in arginine availability for tumour cell growth.

Interestingly, prokaryote defence mechanism *via* increased antimicrobial function was also found to be increased in the progression group. This could result from increase in KOs that are representative of enzymes involved in antimicrobial resistance such as the KO for cationic antimicrobial peptide (CAMP) resistance. This may confer protection for specific pathogens or bacteria against gastric antimicrobial peptides 44 and in turn further alter gastric bacterial composition. Increased bacterial defence could also be achieved *via* increasing stress sensor functions conferred by proteins such as sensor histidine kinases that were found in the progression group 45. This would allow bacteria to adapt to environmental changes such as those from host antitumour response. Further detail analysis identified six specific MetaCyc pathways that were differentially represented and highly related to the previous mentioned metabolic pathways. Of which, biosynthesis of cis-vaccenate which is regulated based on thermal condition of the microbes to control the fatty acid composition of membrane phospholipids 46 was reduced in the progression group. This may be related to the reduction of upstream metabolite in the host or overall changes in microbial composition that has reduced requirement for such processes. On the other hand, TCA cycle VI pathway was also under represented in the progression group. This may suggest a shift in bacterial population (i.e. reduced obligate autotrophs) that is less dependent on aerobic respiration for energy. Interestingly, the chlorophyllide a biosynthesis pathway was enriched in patients that progressed to EGN. Chlorophyllide is a key intermediate molecule during synthesis of chlorophyll and bacteriochlorophylls 47. Several phototropic bacteria including *Chloroflexi*, *Acidobacteria Heliobacteria*, *Chromatium*, *Ectothiorhodospira* and *Chloracidobacterium thermophilum* carry out photosynthesis to produce bacteriochlorophylls as a form of energy 48, 49. Of note, *Chloroflexi* was previously found to be increased in the gastric mucosal of GC patients compared to IM and gastritis 34, which suggests a shift in bacterial diversity in patients that progressed to EGN. Although 16S metagenomic functions were inferred, these results provide insights on the probable microbial pathways attributing to the progression to EGN patients.

## Conclusion

Our study highlights early microbial changes associated with gastric carcinogenesis, whereby *Proteobacteria* microbes (e.g. *Proteus* genus*)* were enriched while *Bacteroidetes* microbes (e.g. *S24-7* family) were depleted in gastric mucosal samples of patients with EGN. We identified a constellation of six microbial taxonomic features present at baseline, which provided the highest classifying power for subsequent EGN. This finding suggests a potential role for prospective microbiome monitoring for GC risk.

## Supplementary Material

Supplementary figures.

Supplementary data 1.

Supplementary data 2.

## Figures and Tables

**Table 1:** Clinical parameters of patients' samples and sequenced map reads output used in this study.

	No IM	Low Risk IM	High Risk IM	Dysplasia	P values
N = 43	17	16	6	4	-
Age (mean (SD))	54.82 (5.00)	62.25 (7.49)	64.33 (10.84)	59.75 (5.74)	0.011
Sex = Male (%)	6 (35.3)	12 (75.0)	5 (83.3)	3 (75.0)	0.055
Clinical history of *H. pylori* infection = Yes (%)	14 (82.4)	9 (56.2)	4 (66.7)	4 (100.0)	0.21
IM grade (%)					< 0.001
Negative	17 (100.0)	0 (0.0)	0 (0.0)	2 (50.0)	-
Mild	0 (0.0)	3 (18.8)	0 (0.0)	0 (0.0)	-
Moderate	0 (0.0)	6 (37.5)	1 (16.7)	0 (0.0)	-
Marked	0 (0.0)	7 (43.8)	5 (83.3)	2 (50.0)	-
Multi focal IM (%)	0 (NaN)	2 (12.5)	6 (100.0)	2 (100.0)	NaN
Transition (%)					< 0.001
Baseline dysplasia	0 (0.0)	0 (0.0)	0 (0.0)	4 (100.0)	-
Developed EGN	1 (5.9)	7 (43.8)	5 (83.3)	0 (0.0)	-
No subsequent EGN	16 (94.1)	9 (56.2)	1 (16.7)	0 (0.0)	-
Map reads (mean (SD))	24246.41 (26665.40)	17889.25 (13308.57)	20217.00 (18521.55)	20051.75 (19164.68)	0.852

## Abbreviations

GC: gastric cancer
IM: intestinal metaplasia
EGN: early gastric neoplasia
OTU: Operational Taxonomic Unit
KO: KEGG orthology
AUC: Area under the ROC Curve
