# Supplementary material for: Mucosal microbiome associates with progression to gastric cancer
Source: Theranostics. 2022 Jan 1;12(1):48–58. doi: 10.7150/thno.65302 (PMC8690935; doi:10.7150/thno.65302)
Supplement: Supplementary file 1 — Supplementary figures. [file thnov12p0048s1.pdf]

Supplementary Figure 1

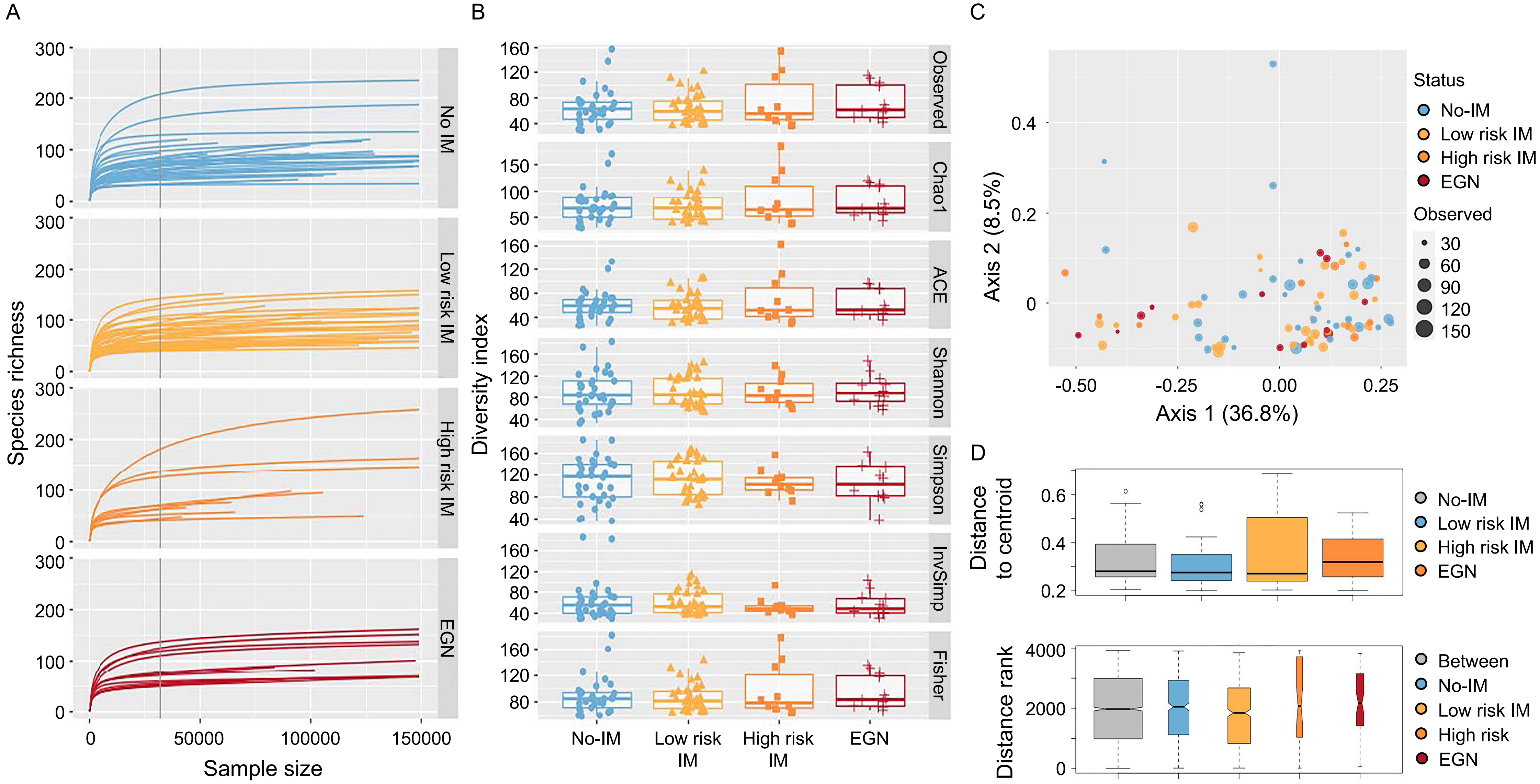

Supplementary Figure 1. Bacterial alpha and beta diversity were comparable between samples from no IM, IM and EGN patients. (A & B) Alpha diversity measurement for species richness and diversity. (C) PCoA of bacterial beta diversity based on the Bray-Curtis dissimilarity. (D) Multivariate homogeneity of groups dispersion analysis based on the Bray-Curtis (Top panel, P = 0.719) and ANOSIM based on Bray-Curtis (Bottom panel, P = 0.209)

Supplementary Figure 2

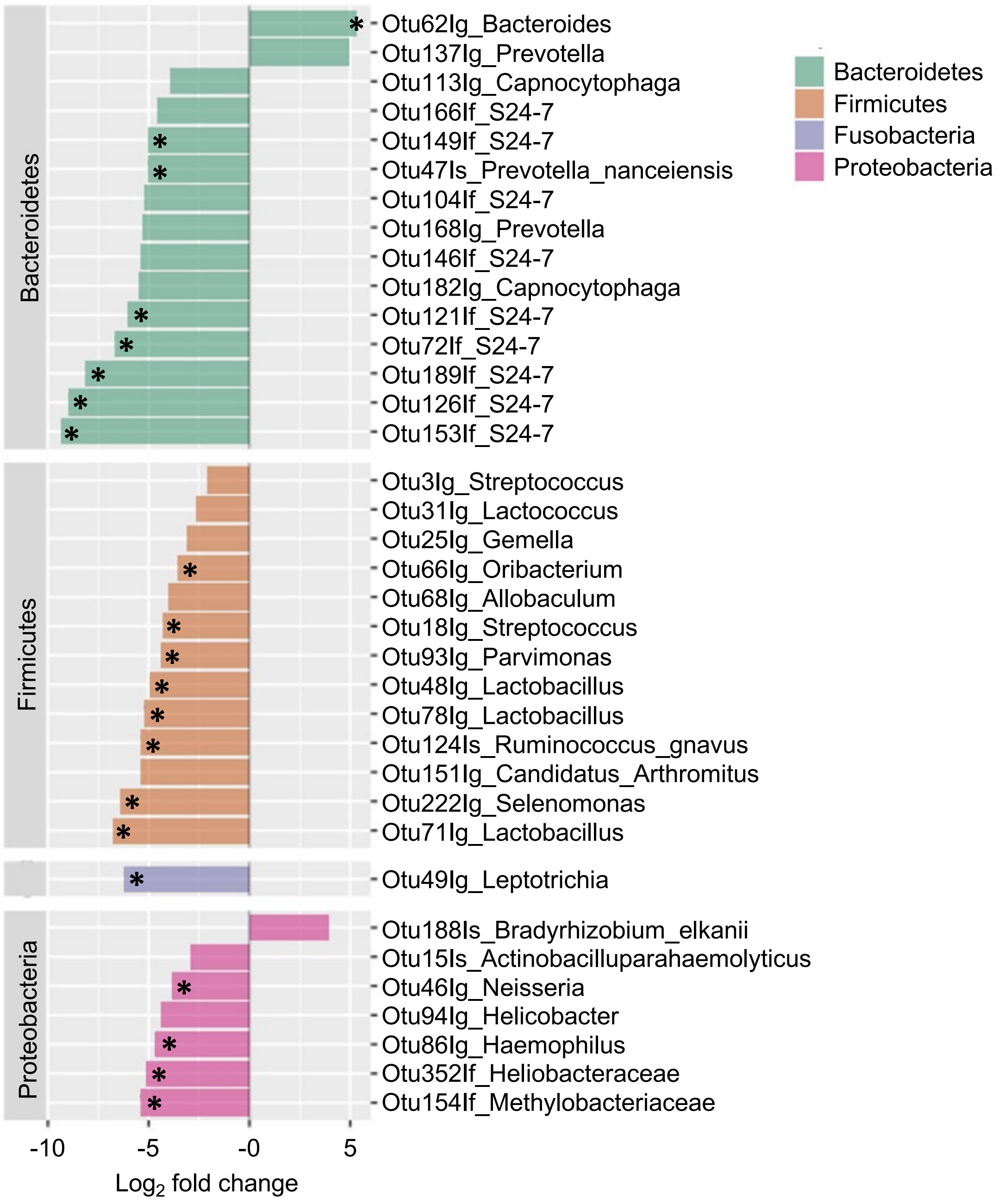

Supplementary Figure 2. Differentially abundant bacterial OTUs in patients that progressed to IM from no IM (baseline) compared to patients with IM regression. Bar chart shows the log2 fold change (X-axis) differences in abundance of top bacterial taxa based on Deseq2 univariant analysis. All comparisons $P < 0.05$, *denotes Benjamini-Hochberg adjusted P (Padj) < 0.1. Of the 20 bacterial OTUs that were significantly associated with IM regression, six were *S24-7*, which is positivity associated with IM regression and negatively associated with IM progression.
